# PEDF-Enriched Extracellular Vesicle for Vessel Normalization to Potentiate Immune Checkpoint Blockade Therapy

**DOI:** 10.34133/bmr.0068

**Published:** 2024-10-01

**Authors:** Sol Shin, Chan Ho Kim, Soyoung Son, Jae Ah Lee, Seunglee Kwon, Dong Gil You, Jungmi Lee, Jeongyun Kim, Dong-Gyu Jo, Hyewon Ko, Jae Hyung Park

**Affiliations:** ^1^ Department of Health Sciences and Technology, SAIHST, Sungkyunkwan University, Seoul 06355, Republic of Korea.; ^2^ School of Chemical Engineering, College of Engineering, Sungkyunkwan University, Suwon 16419, Republic of Korea.; ^3^ Massachusetts General Hospital, Harvard Medical School, Boston, MA, USA.; ^4^ Biomedical Institute for Convergence at SKKU (BICS), Sungkyunkwan University, Suwon 16419, Republic of Korea.; ^5^ School of Pharmacy, Sungkyunkwan University, Suwon, Republic of Korea.; ^6^ ExoStemTech Inc., 55 Hanyangdaehak-ro, Sangnok-gu, Ansan 15588, Republic of Korea.; ^7^ Bionanotechnology Research Center, Korea Research Institute of Bioscience and Biotechnology, Daejeon 34141, Republic of Korea.

## Abstract

The abnormal tumor vasculature acts as the physical and functional barrier to the infiltration and activity of effector T cells, leading to the low response rate of immune checkpoint inhibitors (ICIs). Herein, antiangiogenic extracellular vesicles that enable normalization of the tumor-associated vasculature were prepared to potentiate the efficacy of ICIs. Small extracellular vesicles were exploited as the delivery platform to protect the antiangiogenic protein, pigment epithelium-derived factor (PEDF), from proteolytic degradation. Along with the physicochemical characteristics of the PEDF-enriched extracellular vesicles (P-EVs), their inhibitory effects on migration, proliferation, and tube formation of endothelial cells were investigated in vitro. In tumor-bearing mice, it was confirmed that, compared to bare PEDFs, P-EVs efficiently reduced vessel leakiness, improved blood perfusion, and attenuated hypoxia. Consequently, when combined with anti-PD-1 antibodies, P-EVs remarkably augmented the antitumor immunity, as evidenced by increased infiltration of CD8^+^ T cells and reduced regulatory T cells. These results suggest that P-EVs are promising therapeutics for tumors refractory to ICIs.

## Introduction

Cancer immunotherapy has brought groundbreaking advances in cancer treatment by harnessing the body’s immune system to target and eliminate malignant cells and eliciting durable therapeutic responses [1]. Among the various cancer immunotherapeutic agents, immune checkpoint inhibitors (ICIs) have demonstrated prominent clinical success in many types of cancer [2]. However, only a proportion of patients respond to ICIs, primarily ascribed to immunosuppressive tumor microenvironments (TMEs) [3]. In particular, the aberrant neovasculature of tumor lesions, a hallmark of TME, is a critical bottleneck to immune cell infiltration and function [3,4]. The tumor vasculature is often characterized by immature microvessels with reduced pericyte coverage, increased permeability, impaired blood flow, and endothelial cell anergy. These abnormal features contribute to acidosis and hypoxia in the TME, obstructing effector T cell homing and reinforcing immunosuppression [5]. For instance, the extravasation of CD8^+^ T cells is hindered by overexpressed endothelin receptors and down-regulated adhesion molecules on endothelial cells in TME. Also, expression of Fas ligand (FasL) in the tumor vasculature is associated with scarce CD8^+^ infiltration and a predominance of FoxP3^+^ regulatory T cells (Tregs) [6,7].

In recent years, therapeutic strategies utilizing antiangiogenic agents have gained increasing attention as promising candidates to enhance therapeutic responses of ICIs [5]. Traditional angiogenesis inhibitors, such as antibodies blocking the vascular endothelial growth factor (VEGF) or VEGF receptor, inhibit the formation of new blood vessels or destroy existing vessels to starve tumor cells. Unfortunately, the treatment outcomes were not as effective as expected in clinical settings, and there were also limitations in usage such as developing resistance or promoting metastasis [8,9]. At a low dose of antiangiogenic agents, however, they elicit vascular normalization that transiently repairs tumor vascular abnormalities to improve blood perfusion and alleviate hypoxia, positively affecting the efficacy of various therapeutic regimens [10]. For instance, when combined with vascular normalization strategies, the therapeutic responses of immunotherapies including ICIs were improved, resulting from enhanced infiltration and activation of T cells within the TME.

**Fig. 1.**
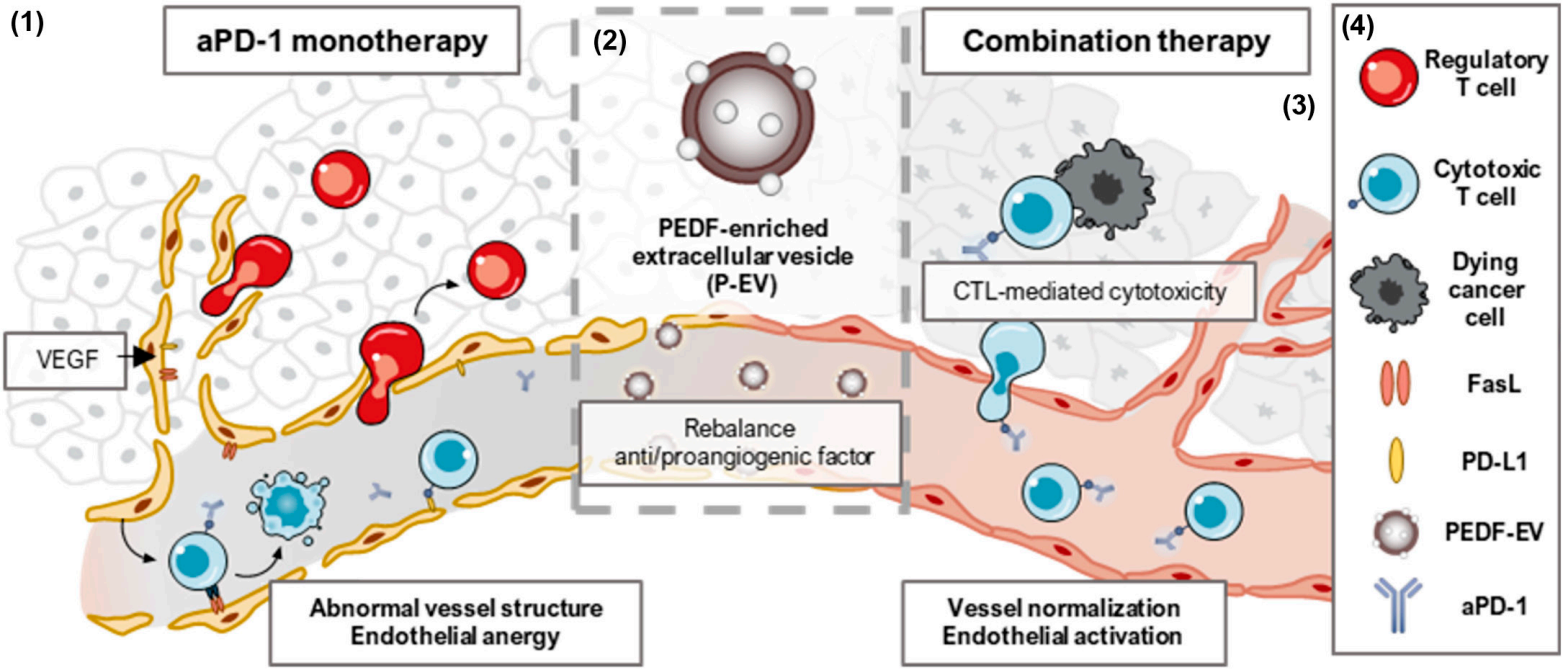
Schematic illustration depicting the mechanism of action of combination therapy using P-EV and aPD-1. P-EV normalizes aberrant tumor vessel and synergistically enhances the antitumor effect of aPD-1 as follows: (1) A relentless exposure to VEGF, secreted from the tumor, renders endothelial cells being in a constant activation state, leading to formation of an abnormal vessel that hinders T cell activation and infiltration into the tumor. (2) P-EV suppresses VEGF signaling in tumor tissue, (3) transiently repairs tumor vascular abnormalities, (4) increases infiltration of CD8^+^ T cells, and reduces Tregs.

Pigment epithelium-derived factor (PEDF), a promising candidate as an antiangiogenic agent, is a 50-kDa secreted glycoprotein and a member of the serine protease inhibitor (serpin) superfamily that was first identified as a neurotrophic factor. Its therapeutic potentials, however, have been limited by susceptibility to proteolytic degradation, resulting in a short biological half-life [8,11]. Motivated by the prospect of vascular normalization to improve cancer immunotherapy, we herein exploited the small extracellular vesicles (EVs), as the carrier of PEDF. EVs, secreted by various cell types, are membrane-bound vesicles with a size of 50 to 200 nm and play essential roles in intercellular communication by transporting diverse cargo (i.e., proteins, nucleic acids, lipids, etc.) from parent cells to recipient cells [12]. Their inherent property to cross biological barriers and shield cargo molecules, including proteins like PEDF, from degradation makes them attractive drug carriers [13]. Given the biocompatibility and potential of EVs as the delivery platform, PEDF-enriched EVs (P-EVs) could be the promising option for combination therapy with ICIs by eliciting the optimal activity of PEDF and reconstituting the TME (Fig. 1).

To investigate this hypothesis, P-EVs were isolated from preconditioned cells. The preconditioning approach offers several advantages for engineering EVs with desired target molecules, including ensuring the proper incorporation of cargo without compromising their native structures. Efforts to improve the functional and clinical utility of EVs have mainly focused on refining drug loading methods [14,15]. However, many of these strategies involve complex procedures such as gene transfection or postisolation loading methods that can adversely affect the structural integrity of EVs. In contrast, our study aims to modulate the TME by using functional EVs that overexpress specific proteins through an efficient preconditioning method. This method mitigates potential toxicity and simplifies the modification process, making it a safer and more practical alternative for clinical applications. In the present study, we confirmed the biological activity of P-EVs by evaluating their ability to inhibit migration, proliferation, and tube formation in endothelial cells. The promising aspect of P-EVs was further identified in vivo by assessing whether the therapeutic responses of anti-programmed death-1 antibodies (aPD-1) and antitumor immunity can be improved by vascular normalization.

## Materials and Methods

### Materials

Tris base, Triton X-100, sodium chloride (NaCl), Tween 20, bovine serum albumin (BSA), β-mercaptoethanol, nonessential amino acid solution, thiazolyl blue tetrazolium bromide (MTT), and Giemsa stain solution (Cat no. 51811) were obtained from Sigma-Aldrich (MO, USA). Radioimmunoprecipitation assay lysis buffer, Halt protease inhibitor cocktail, micro bicinchoninic acid protein assay kit, and Pierce ECL western blotting substrate (32109) were purchased from Thermo Fisher Scientific (MA, USA). Recombinant Human SERPINF1 / PEDF was obtained by LS Bio (WA, USA). Dulbecco’s modified Eagle medium (DMEM), RPMI 1640 medium, fetal bovine serum (FBS), Dulbecco’s phosphate-buffered saline (DPBS), penicillin/streptomycin (100×), and trypsin-EDTA were purchased from Hyclone (CA, USA).

### Cell line and animals

Cell lines were purchased from the Korean Cell Line Bank (Seoul, Republic of Korea) unless otherwise specified. 4T1 murine mammary carcinoma (American Type Culture Collection, Manassas, VA) and C166 murine endothelial cell lines were cultured in RPMI 1640 containing 10% FBS and 1% penicillin/streptomycin. Primary human umbilical vein endothelial cells (HUVECs, #C-12200) and endothelial growth media 2 (EGM2, #C-22011) were obtained from PromoCell (Heidelberg, Germany). HUVECs were cultured in EGM2-containing supplements and 1% antibiotic–antimycotic. For all in vitro experiments, cells were cultivated at 37 °C in a humidified incubator with 5% CO_2_.

For in vivo experiments, 5- to 6-week-old female Balb/c mice were purchased from Orient Bio Inc. (Seongnam, Republic of Korea) and maintained under specific pathogen-free conditions. All experiments involving live animals were conducted in compliance with relevant ethical regulations and protocols approved by the Institutional Animal Care and Use Committee of Sungkyunkwan University.

### Human adipose tissue-derived stem cell culture and cell preconditioning

Primary human adipose tissue-derived stem cells (hADSCs) (#C-12971), purchased from Cefobio Inc. (Seoul, Korea), were cultured in DMEM supplemented with 10% FBS and 1% penicillin/streptomycin at 37 °C in humidified air containing 5% CO_2_. Before preconditioning, hADSCs were seeded at 5,000 cells cm^−2^ and incubated to reach 100% confluence. hADSCs were incubated for 21 d in a preconditioning medium containing 100 nM dexamethasone and 45 μM (+)-sodium L-ascorbate and β-glycerophosphate disodium. The fresh medium was replaced every 3 d. Conditioned medium (CM) was replaced to isolate EVs at various preconditioning timespans (0, 1, 3, 7, 14, and 21 d). The EVs were denoted as EV^xd^, where xd indicates the preconditioning period in day.

### EV isolation

EVs were isolated from CM via serial centrifugation and filtration. CM was collected after preconditioning hADSC for 21 d. Briefly, Cells were washed with DPBS, and their media were changed with CM for 24 h. Collected CM was centrifuged (20 min, 3,000 rpm) to deplete cell debris and filtrated through a 0.22-μm membrane to remove the cell fragments. EVs were then isolated using a tangential flow filtration capsule (300-kDa molecular weight cutoff ultrafiltration membrane filter; Pall Corporation, Port Washington, NY, USA), followed by dispersion in PBS. EVs, isolated from proliferating hADSC (C-EV), were used as a control.

### Characterization of EVs

The particle size and distribution of EVs were measured using the nanoparticle tracking analysis (NTA) system (NanoSight LM10, Malvern Instruments, England), as described previously [16]. The NTA measurements were set as follows: capture duration, 30 s; shutter speed, 30 ms; viscosity, 1.0 c; camera level, 16; detection threshold, 5; screen gain, 10. The morphology of EV was observed using a transmission electron microscope (TEM) (JEM-2100F, JEOL Ltd., Tokyo, Japan) operating at 200 kV. For TEM sample preparation, EVs were fixed with a 2% paraformaldehyde solution for 5 min, dropped on a 200-mesh carbon-film-coated grid, negatively stained with 1% uranyl acetate for 1 min, and washed with water. The PEDF content on EVs was determined using a Human PEDF enzyme-linked immunosorbent assay (ELISA) kit (Abcam, Cambridge, UK), following the manufacturer’s instructions.

### Western blot analysis

Proteins were separated by 8% sodium dodecyl sulfate-polyacrylamide gel electrophoresis. Anti-CD9 antibody (1:200), anti-calnexin antibody (1:5,000), anti-GM130 antibody (1:200), anti-TSG101 antibody (1:200), and anti-β-actin antibody (1:2,000) were used as primary antibodies. Horseradish peroxidase-conjugated anti-rabbit immunoglobulin G (IgG) (1:500) or anti-mouse IgG (1:1,000) was used as the secondary antibody. The membranes were examined using LAS-3000 (Fuji Photo Film, Japan), and the molecular weights of the proteins were distinguished using a protein ladder (Precision Plus Protein Dual Color Standards; BIO-RAD Laboratories, Hercules, CA, USA).

### EV labeling and in vitro cellular uptake

The fluorescent EVs were freshly prepared using a Cy5.5-NHS ester (Lumiprobe, MD, USA). In brief, 200 μg of EVs were suspended in DPBS (pH 7.4) and mixed with a Cy5.5-NHS solution in dimethyl sulfoxide. The reaction was conducted at 4 °C overnight under continuous stirring. The reaction mixture was purified using gravity flow columns (PD-10 Desalting columns; GE Healthcare, USA) to remove unreacted free dyes. Thereafter, the labeled EVs were incubated in the dark condition with serum-free HUVECs at the seeding intensity of 2 × 10^4^ cells cm^−2^ in 24-well plates for 1, 3, 6, or 12 h. The preparations were then fixed with 4% paraformaldehyde for 30 min, and the cell nucleus was stained with 6-diamidino-2-phenylindole (DAPI, Sigma, USA). Images were obtained using a confocal microscope (Leica Microsystems, Wetzlar, Germany).

### In vitro cell migration assay

For all in vitro cell studies, experimental groups were as follows: C-EVs (100 μg ml^−1^), recombinant PEDF proteins (PEDF-L; 20 ng ml^−1^, PEDF-H; 100 ng ml^−1^), and P-EVs (100 μg ml^−1^, equal amount of PEDF as PEDF-L).

For assessment of cell migration, HUVECs were plated at a density of 2 × 10^4^ cells in the upper chamber of the transwell plate fitted with an 8-μm pore membrane (Corning Inc., Corning, NY). To investigate the effect of EVs on inhibiting cell migration, the upper chamber was loaded with EVs (100 μg ml^−1^). Then, complete EGM2 was added to the lower chamber as a chemoattractant. Nonmigrated cells were removed at predetermined time points from the upper surface, and filters were stained with Giemsa. Images were obtained using an inverted microscope (Nikon Eclipse Ti-U microscope, Tokyo, Japan), and the number of migrated cells was quantified using an ImageJ software.

We also measured the number of migrated cells in the experimental setting to mimic extravasation of cancer cells at primary tumors by coincubating 4T1 cells. A total of 5 × 10^4^ 4T1 cells were loaded in the upper chamber while suspended in 200 μl of serum-free DMEM medium in presence of EVs. At 24 h, the inner surfaces of the upper chambers were wiped using a cotton swab to remove nonmigrated cells, and the membranes were detached for mounting on glass slides and nuclei were stained using DAPI Fluoromount-G.

### In vitro cell proliferation assay

Cell proliferation behaviors of HUVECs were evaluated using the MTT assay. Briefly, cells were seeded in a 24-well plate at a density of 2 × 10^4^ cells well^−1^ and grown for 24 h. Then, the culture medium was replaced with a fresh medium containing 2% FBS and P-EV or C-EV for another 24 h, and 48 h. At the end of the incubation, the MTT reagent was added to each well and incubated for 3 h at 37 °C. After removal of the MTT-containing supernatant solution, dimethyl sulfoxide was added to each well to dissolve violet formazan crystals. Thereafter, the optical density of each well was assessed with a microplate reader at 570 nm.

### In vitro tube formation assay

For the capillary-like tube formation assay, growth factor-depleted Matrigel (BD Pharmingen, CA, USA) was applied to a 24-well plate (250 μl per well). After polymerization of the Matrigel at 37 °C for 1 h, HUVECs starved for 2 h were harvested and resuspended in an assay medium (with supplement, 100 U ml^−1^ penicillin, and 80 U ml^−1^ streptomycin). Then, HUVECs (2 × 10^4^ cells ml^−1^) were added to each chamber and incubated for 24 h at 37 °C in 5% CO_2_, followed by imaging of capillary-like tube formation using phase-contrast microscopy. The capillary network was analyzed by calculating the cumulative number of tubes in 3 random microscopic fields using computer-assisted microscopy.

### In vivo antitumor efficacy and antimetastasis testing

To evaluate the possible effect of P-EVs in vivo, Balb/c mice were subcutaneously inoculated with 1 × 10^6^ 4T1 cells per mouse. When the average tumor volume reached 250 mm^3^, the tumor-bearing mice were randomly divided into each treatment group as follows: DPBS, C-EV (10 μg head^−1^), P-EV (10 μg head^−1^), and recombinant protein PEDF (PEDF-H, 10 ng head^−1^). The mice were administered intratumorally with reagents in a 50-μl volume. The tumor volumes and body weights were recorded daily. Tumor tissues were obtained for analysis at 15 and 18 d after tumor inoculation.

To evaluate antitumor efficacy of combination therapy, 5 × 10^5^ 4T1 cells were suspended in a Matrigel solution (50 μl) and subcutaneously implanted into the third mammary pad of Balb/c mice. When the average tumor volume reached 75 cm^3^, the tumor-bearing mice were randomly divided into each treatment group: DPBS, aPD-1 (Clone RMP1-14, Cat no. BE0146, InVivoMAb), P-EV, and P-EV+aPD-1. The mice were administered intratumorally with reagents in a 50-μl volume and intraperitoneally with 5 mg kg^−1^ aPD-1 in a 100-μl volume. P-EV treatments were given 2 times before antibody treatment commenced. The tumor volumes and body weights were recorded daily and calculated as follows: volume (mm^3^) = 0.5 × length (mm) × width (mm) × width (mm). After administrations were finished, the mice were euthanized to collect tumor tissues and relevant organs for further analysis. After sacrifice, lungs and livers were harvested for antimetastasis testing. The collected organs were fixed with 10% neutral buffered formalin and further processed for histological examination. The metastatic lesions were analyzed by hematoxylin and eosin staining and imaged using an Axio scan Z1 slide scanner (Zeiss, Oberkochen, Germany).

### Power Doppler ultrasound imaging of tumors

Power Doppler ultrasound imaging of tumors was performed as described previously [17]. Tumors were imaged using a photoacoustic and high-frequency microimaging platform (Vevo LAZR-X, Fujifilm VisualSonics, CA, USA). Tumor-bearing mice were anesthetized to minimize echogenicity attributable to tissue motion. The ultrasound transducer, used for power Doppler imaging, transmitted a central frequency of 32 MHz with a focal length of 6 mm. An acoustic standoff between transducer face and tumor was achieved by the application of acoustic gel. Initial scanning of each tumor was performed in B-mode (grayscale ultrasound) to define the boundary of the tumor mass based on echogenicity parameters. A rectangular area was then placed around the tumor and surrounding tissue, denoting the region in which power Doppler data would be acquired. For analysis, a region of interest was drawn around the tumor boundary based on the initial B-mode scan. The mean color level, which is the average intensity of the Doppler signal within a tumor region of interest, was used to estimate blood flow.

### In vivo blood vessel perfusion and leakiness analysis

On days 15 and 18 after tumor inoculation, 6 mice were randomly selected from each group. Tumor perfusion was quantified on tumor cryosections after intravenous injection of fluorescein (FITC)-labeled *Lycopersicon esculentum* (tomato) lectin (50 μg, FL-1171, Vector Laboratories) into tumor-bearing mice 10 min before tumors were harvested for analysis. Tumor vascular leakage was evaluated by intravenous injection of 1 mg of FITC-labeled dextran (40 kDa; FD40S Sigma-Aldrich) into tumor-bearing mice 30 min before harvesting tumors. Then, immunofluorescence staining was performed for the quantification of FITC–lectin^+^CD31^+^ vessels or FITC–dextran^+^CD31^+^ vessels.

### Immunohistochemistry

Immunohistochemistry was performed on either tissue cryosections or paraffin-embedded tissue sections. The tissue cryosections were fixed in ice-cold acetone and blocked for 1 h with DPBS containing 1% BSA. For paraffin-embedded tissue sections, an antigen-retrieval step was conducted by steaming in citrate buffer (pH 6.0), and the sections were blocked for 1 h with DPBS containing 1% BSA. The primary antibodies were diluted with staining buffer (DPBS with 1% BSA and 0.1% Triton X-100; PBS-T), according to the manufacturer’s instructions. The sections were incubated with primary antibodies overnight at 4 °C and washed 3 times with PBS-T. The sections were further stained with secondary antibodies for 1 h at room temperature. The following antibodies were used: anti-CD8-Alexa Fluor 594 (clone 53-6.7, BioLegend), anti-α-SMA-FITC (clone 1A4, Invitrogen), anti-fibronectin (Cat no. ab2413, Abcam, Cambridge, UK), anti-CD31 (PA5-16301, Invitrogen), anti-hypoxia inducible factor 1α (HIF-1α) (clone S100A4, BioLegend), and goat anti-Rabbit IgG-Alexa Fluor 594 (Cat no. ab150080, Abcam). After staining nuclei with DAPI Fluoromount-G, images were obtained using a confocal microscope.

### Statistical analysis

Data were analyzed with a GraphPad Prism software. Efficacy data were compared by one-way analysis of variance (ANOVA) with Tukey’s multiple comparisons test and reported as multiplicity-adjusted *P* values. All data are presented as mean ± SD. We indicated the significant difference between datasets as follows: *P < 0.05, **P < 0.01, ***P < 0.001, ****P < 0.0001, and *ns*, not significant.

## Results

### Preparation and characterization of PEDF-enriched EVs

Recently, EVs have gained significant attention for tissue remodeling in the field of cancer research [18]. In this study, we aimed to investigate the potential of PEDF-overexpressed EVs, derived from preconditioned parent cells, as modulators of the TME. The increased intracellular synthesis of PEDF has been reported when mesenchymal stem cells were cultured in an osteogenic medium [19]. Based on this premise, we assessed whether such a condition could be adapted to produce P-EVs from hADSCs. First, hADSCs were cultured in a preconditioning medium supplemented with dexamethasone, (+)-sodium L-ascorbate, and β-glycerophosphate disodium. Then, EVs were isolated from serum-free medium collected at various preconditioning timespans (EV^0d^, EV^1d^, EV^3d^, EV^7d^, EV^14d^, EV^21d^, and EV^28d^). As expected, all EV samples consistently had similar sizes (~150 to 190 nm in diameter) with round shapes (Fig. S1A and B). Meanwhile, the number of EVs and their total protein amount per unit volume showed an increasing trend over preconditioning up to 28 d (Fig. S1C and D). Notably, ELISA and western blot analysis confirmed that EV^21d^ expressed the highest PEDF when normalized to the corresponding total protein concentration of EV (Fig. S1E). Considering the above experimental results, EV^21d^, referred to as P-EV, was chosen for further experiments. We also isolated EVs from hADSCs in nontreated media to prepare isotype control EVs (C-EVs).

**Fig. 2.**
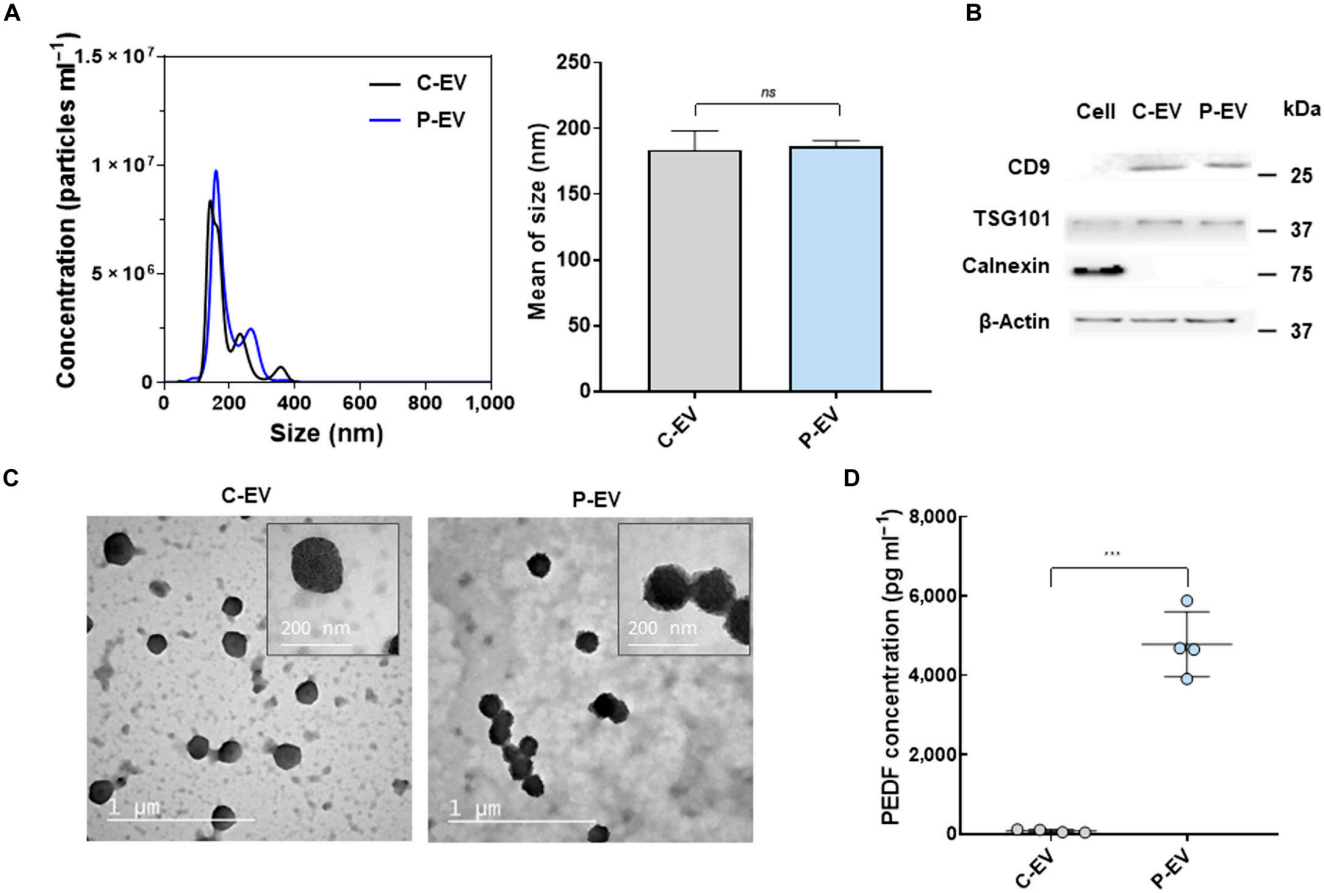
Characterization of P-EV. (A) Size distribution and average mean size of C-EVs and P-EVs by NTA and DLS. (B) Western blot analysis of EVs for EV markers (CD9 and TSG101) and the non-EV marker (Calnexin). (C) Representative TEM images of C-EVs and P-EVs. Scale bar, 1 μm and 200 nm. (D) Expression level of PEDF on C-EVs and P-EVs, measured by ELISA (*n* = 4). Significance was determined using an unpaired one-tailed *t* test. ***P < 0.001. Error bar, SD.

As depicted in Fig. 2A, both C-EVs and P-EVs had similar size distributions and exhibited a negligible size discrepancy with an average size of 183.7 and 186.7 nm, respectively. Western blotting confirmed the presence of EV-specific markers (CD9 and TSG101) and the absence of non-EV features (Calnexin) in both EV samples (Fig. 2B). Also, their typical round spheroid morphology was observed in the wide-field and close-up TEM images (Fig. 2C). These results validated the successful isolation of C-EVs and P-EVs. Furthermore, the antiangiogenic PEDF level of P-EVs was verified to be significantly higher (60-fold) than that of C-EVs, as quantified by ELISA (Fig. 2D).

### P-EV inhibits angiogenesis in vitro

**Fig. 3.**
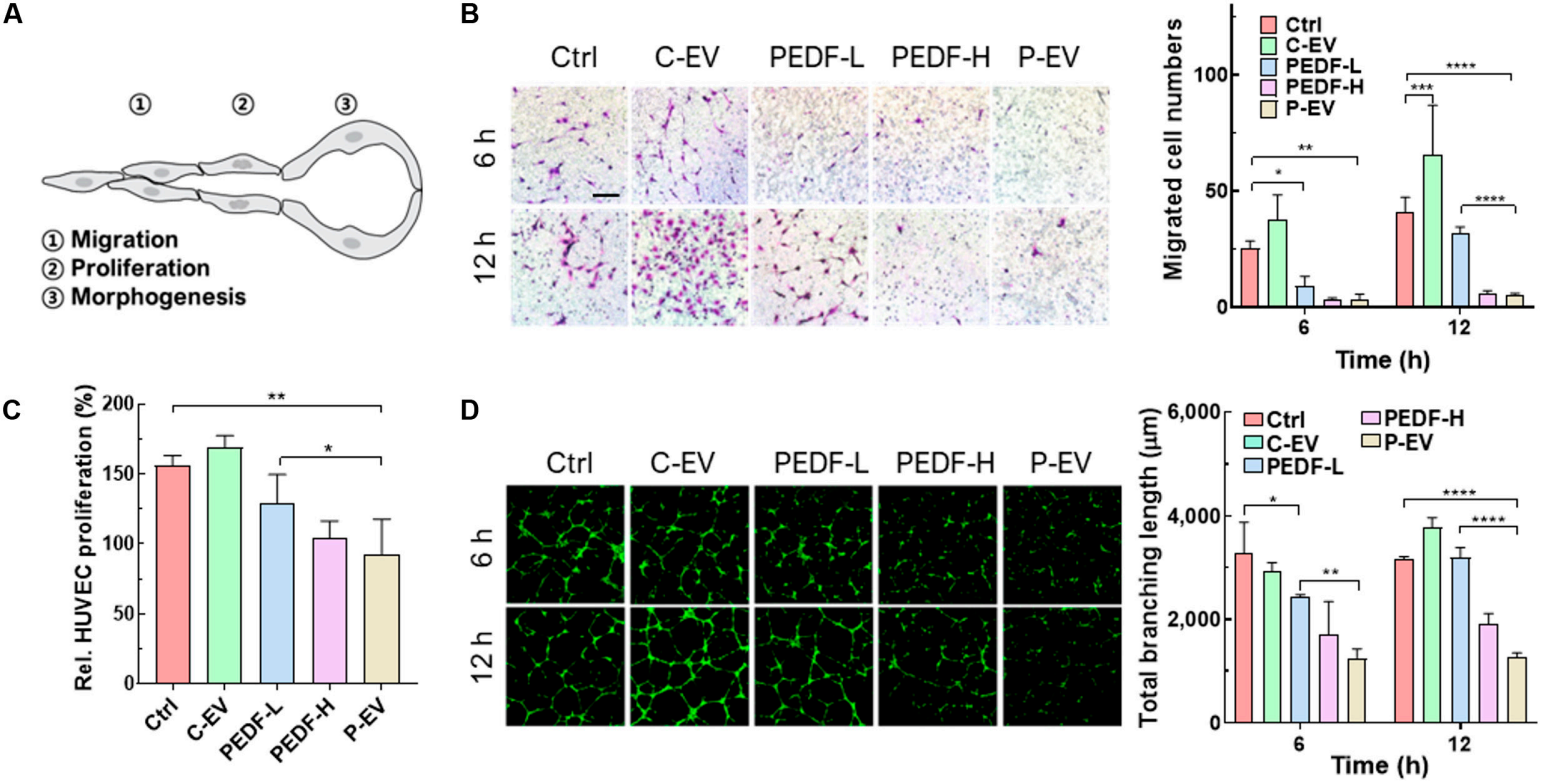
P-EV inhibits angiogenesis in vitro. (A) Illustration of sequential events in angiogenesis. (1) Basement membrane disintegration opens the way for endothelial cell (EC) migration. (2) ECs proliferate and (3) undergo morphogenesis involving formation of lumen-bearing cords. (B) Transwell assay to assess the effect of P-EV on migration of HUVECs. Migrated cell images (left) and quantification of migrated cells (right). (C) Proliferation of HUVECs. (D) Representative images and quantitative analysis of tube formation.

The PEDF proteins block the VEGF signaling pathways, ultimately suppressing the relevant endothelial responses [11]. Before thoroughly investigating the antiangiogenic potential of P-EVs with HUVECs, confocal microscopy experiments were performed to observe their cellular uptake behaviors involving cell receptor binding (Fig. S2) [20]. Fluorescence images confirmed that P-EVs and C-EVs exhibited increased fluorescence signals with similar uptake trends for HUVECs over time. Then, their impact on the angiogenic cascade of endothelial cells, which includes proliferation, migration, and morphogenesis, was evaluated (Fig. 3A). For the in vitro cell experiments, HUVECs were treated with C-EVs, PEDF-L, PEDF-H, and P-EVs. It should be noted that the P-EVs contain 5 times less PEDF than PEDF-H and an equal amount of PEDF as PEDF-L. The migration of HUVECs was assessed using a transwell assay with the number of migrated cells calculated (Fig. 3B). The results showed that, compared to the control, migration of the HUVECs slightly increased by time in the C-EV-treated group and somewhat decreased only at the early time point of 6 h in the PEDF-L-treated group. Interestingly, the PEDF-H- and P-EV-treated groups remarkably inhibited cell migration even at the 12-h time point by 86.06% and 87.70%, respectively, with no significant differences between these 2 groups. In the proliferation assay, the control, C-EVs, and PEDF-L promoted the proliferation of HUVECs to a similar extent, whereas PEDF-H and P-EVs significantly inhibited their proliferation (Fig. 3C). Additionally, the disruption of endothelial network formation was examined using a capillary-like tube formation assay, where HUVECs were plated onto Matrigel and treated with samples for 6 or 12 h. Microphotographs were obtained to visualize their effects on tube formation (Fig. 3D). The number of capillaries in both PEDF-H- and P-EV-treated groups greatly decreased over the other groups, where normal tube structures were destroyed with interrupted alignments and cords. Treatment with PEDF-L also marginally reduced the number of cord formations at the early time point of 6 h, but the tube structures observed in this group at the 12-h time point were as regular as those in the control group. The tube formation was quantitatively analyzed with total branching length (Fig. 3D), the mean mesh size, and the number of segments, branches, and junctions per image (Fig. S3). C-EVs did not disrupt the tube formation and exhibited structures similar to those of the control group, indicating their lack of ability to inhibit angiogenesis without PEDF. In contrast, the quantification data were consistent with the microscopy images, highlighting the superior antiangiogenic capability of PEDF-H and P-EVs to the other samples. Representatively, PEDF-H and P-EVs significantly reduced total branching length to 1,902.3 ± 173.1 μm and 1,268.6 ± 67.7 μm, respectively, compared to the control (3,156.7 ± 45.6 μm) at 12-h time point. Overall, PEDF-H and P-EVs demonstrated comparable PEDF-mediated antiangiogenic properties in modulating endothelial cell behaviors. Conversely, C-EVs, lacking PEDF, did not exhibit significant antiangiogenic activity and showed angiogenic processes similar to those observed in the control group. This suggests that the PEDF in P-EV exhibits enhanced biological activity compared to its free soluble form, considering that the amount of PEDF in P-EVs is 5 times less than in PEDF-H, and the action of PEDF leads to angiogenesis inhibition.

### P-EV delays tumor growth and reduces tumor hypoxia in mice

**Fig. 4.**
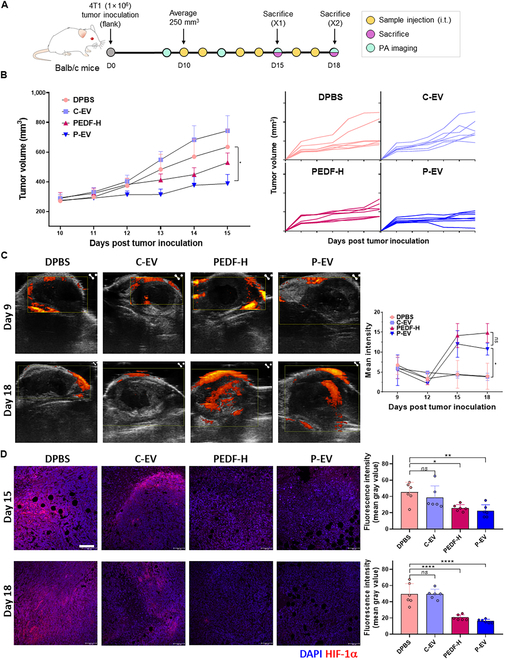
P-EV delays tumor growth and reduces tumor hypoxia in mice. (A) Schematic illustration of the therapeutic schedule for tumor-bearing mice. 4T1 tumor-bearing Balb/c mice were treated with EVs (10 μg head^−1^) on selected days postinoculation. (B) Tumor volumes were measured starting at 10 d postinoculation and results are reported as mean ± SD (left, one-way ANOVA). Individual tumor volume (right). (C) Power Doppler ultrasound images of tumors at 9 and 18 d after tumor inoculation. Representative images are shown for the same tumor with B-mode images (grayscale) overlaid with power Doppler (red). (D) Immunofluorescence microscopy images of HIF-1α and CD31 in tumor tissues. Scale bar, 100 μm. Blue, cell nuclei. The representative sections of 3 independent experiments were repeated. Significance was determined using one-way ANOVA. ****P < 0.0001, ***P < 0.001, **P < 0.01, and *P < 0.05. Error bar, SD.

Based on the prominent antiangiogenic activity of the small EV-PEDF (ePEDF) on HUVECs, we next investigated the in vivo beneficial effects of P-EV on cancer therapy using the murine flank tumor model. Mice were inoculated with 4T1 cells, and treatment regimens began with P-EVs, comparable recombinant protein (PEDF-H), and control groups (DPBS and C-EVs), according to the administration schedule summarized in Fig. 4A. As indicated by negligible changes in mice body weight, all treatment regimens were well tolerated (Fig. S4A). While the tumor volume of the entire group continued to increase over time, its growth showed a statistically significant delay in P-EV-treated mice compared to the control groups except for PEDF-H (Fig. 4B). The anticancer effect of P-EV monotherapy may be attributed to its positive impact on tumor vascular integrity resulting in increased intratumoral blood perfusion and reduced hypoxia, as seen with other antiangiogenic drugs [21]. Thus, power Doppler ultrasound image analysis was performed for tumor tissues, taken at 9, 12, 15, and 18 d after tumor inoculation, indicating that both PEDF-H and P-EVs significantly increased global vessel perfusion at 15 and 18-d time points over the control (Fig. 4C). This aligns with the fact that during treatment with antiangiogenic agents, there is usually a time window when the structure and function of tumor vessels become transiently normal [22]. Moreover, immunofluorescence staining of tumor tissues revealed lower expression of HIF-1α in the PEDF-H- and the P-EV-treated groups than controls at both 15 and 18-d time points, demonstrating their significantly reduced intratumoral hypoxia (Fig. 4D). These notable changes, observed in the TME of mice treated with either PEDF-H or P-EVs, agree with the findings from the PEDF experiment, documented by Xu et al. [23]. Meanwhile, consistent with the in vitro cell study results, the overall in vivo performance of the PEDF-H and P-EV groups was comparable. Recalling 5 times less PEDF contents in P-EV than PEDF-H, the biological activity of PEDF was significantly improved when loaded onto EVs in this study, highlighting the potential benefits of EV-mediated delivery in enhancing its therapeutic efficacy within the complex TME [18]. This is probably because ePEDF is mainly located on the outer surface of EV, making it more effective than recombinant PEDF in interacting with receptors of target cells to mediate the biological events involved [24].

### P-EV improves the antitumoral activity of aPD-1

**Fig. 5.**
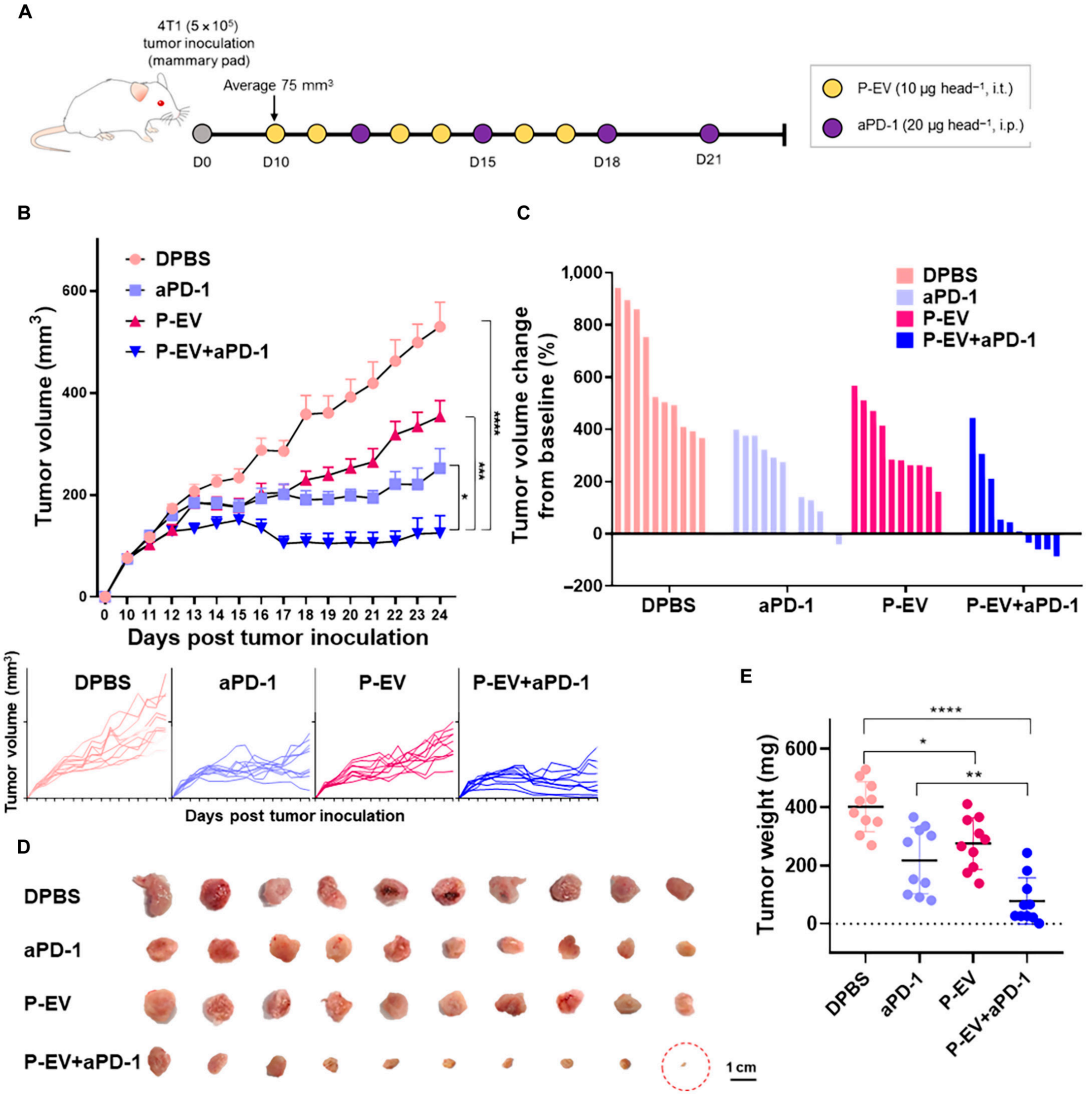
P-EV synergistically enhances the antitumor effect of an ICI. (A) Schematic illustration of the therapeutic schedule for tumor-bearing mice. 4T1 tumor-bearing Balb/c mice were treated with P-EV (10 μg head^−1^), aPD-1 (20 μg head^−1^), or combinations thereof on selected days postinoculation. (B) Tumor volumes were measured starting at 10 d postinoculation and results are reported as mean ± SD Individual tumor volume (bottom). (C) Tumor volume change of individual mice from the initial volume. (D) Photographs of the tumors harvested on day 24 (*n* = 10). (E) Tumor weight (*n* = 10). Significance was determined using one-way ANOVA. ****P < 0.0001, ***P < 0.001, **P < 0.01, and *P < 0.05. Error bar, SD.

In the context of immune checkpoint blockade therapy, vascular dysfunction contributes to immunotherapeutic resistance [7], which motivated us to explore whether P-EV treatment prior to aPD-1 injection could enhance the therapeutic outcome of an aPD-1. Thus, we evaluated the antitumor efficacy of P-EV and aPD-1 combination therapy in the murine orthotopic breast cancer model. When the average tumor volume reached 75 cm^3^, treatment regimens began with DPBS, P-EV, aPD-1, or P-EV with aPD-1 (P-EV+aPD-1) according to the administration schedule summarized in Fig. 5A. After measuring the tumor volume for 24 d, its change from baseline for each mouse was calculated to present the tumor inhibition rate, followed by photographing and weighing excised tumor masses (Fig. 5B to E). Taken all together, P-EV itself showed a statistically insignificant impact on tumor growth, compared to aPD-1 monotherapy. In marked contrast, the superior antitumor efficacy of P-EV+aPD-1 combination therapy was clearly demonstrated with reduced tumor volume and weight by 50.45% and 60.06%, respectively, compared to the aPD-1 treatment alone. During the treatment period, the body weight of mice remained within the normal range, indicating that all treatments were safe in vivo (Fig. S4B). We then focused on the effect of antiangiogenic P-EVs on tumor metastasis. Because tumor hypoxia and aberrant vessel promote metastasis by increasing the extravasation of cancer cells [25], the previously demonstrated improvement of tumor oxygenation and vascular integrity by P-EVs may contribute to decrease cancer cell dissemination. While micrometastases were led in both organs, fewer hepatic and pulmonary metastatic nodules were observed in both P-EV-treated groups than in the other groups (Fig. S5). The results indicated that P-EV treatment not only modified tumor proliferation but also prevented the metastasis-promoting functions of VEGF in vivo.

### P-EV normalizes tumor vessels and increases infiltration of CD8^+^ immune cells

**Fig. 6.**
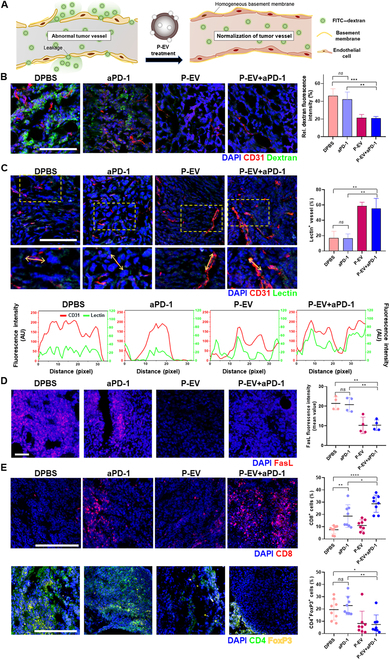
P-EV normalizes tumor vessels and increases tumor infiltration of immune effector cells. (A) Schematic illustration of the permeability assay. Administration of FITC–dextran into tumor vessels allows visualization of their barrier function. Immature blood vessels exhibit a defective barrier function, leading to the leakage of FITC–dextran. (B) Representative immunofluorescence microscopic images of CD31 and dextran in tumor tissues. Scale bar, 100 μm. Quantification of FITC–dextran leakage in TME (right, *n* = 3). (C) Representative immunofluorescence microscopic images of CD31 and lectin in tumor tissues. Scale bar, 100 μm. Quantification of lectin-labeled vessel in TME (right, *n* = 3). Kinetic quantification of confocal microscopic images (bottom). (D) Representative immunofluorescence microscopic images of FasL in tumors (left). Scale bar, 50 μm. Quantification of FasL expression (right, *n* = 4). (E) Representative immunofluorescence microscopic images of CD8^+^ cells (top) and CD4^+^FoxP3^+^ cells (bottom) in TME. Scale bar, 200 μm. Quantification of CD8^+^ cells or CD4^+^FoxP3^+^ cells in TME (right, *n* = 8). Significance was determined using one-way ANOVA. ****P < 0.0001, ***P < 0.001, **P < 0.01, and *P < 0.05. Error bar, SD.

To explore the mechanistic basis for the improved antitumor efficacy of P-EV+aPD-1 combination therapy, we first investigated the effect of P-EV on normalization of tumoral vessels. After intravenous injection of FITC–dextran into tumor-bearing mice, CD31 and FITC–dextran were observed to estimate endothelial vessel leakiness in vivo (Fig. 6A). Both P-EV and P-EV+aPD-1 significantly reduced the dextran-positive leakage area of tumors compared to DPBS and aPD-1 groups (Fig. 6B). Since vascular leakage can lead to defects in vessel perfusion [11], FITC–lectin was further applied to visualize and analyze normalized basal membrane of tumor endothelium. Interestingly, perfused lectin^+^CD31^+^ tumor microvessels were abundant in P-EV-treated mice, whereas only a few blood vessels were lectin^+^ in the other groups (Fig. 6C). The contribution of P-EV to reduced dextran leakage and enhanced vessel perfusion indicates that ePEDF effectively improved tumor vessel integrity and functionality. We also observed tumor endothelium-specific FasL, which is responsible for regulating the balance between CD8^+^ T cells and Tregs, as documented in previous studies [7,26]. FasL expression is relatively specific to endothelium in primary and metastatic tumors, with tumor cells themselves expressing no or low levels of FasL [27]. Moreover, evidence suggests that VEGF regulates FasL expression on the tumor endothelium, leading to selective apoptosis of CD8^+^ T cells while sparing Tregs. This inverse association with CD8^+^ T cell infiltration underscores the vessel FasL expression [26]. Both P-EV-containing therapies reduced VEGF-induced FasL expression (Fig. 6D). Conversely, other groups exhibited a substantial FasL expression in the blood vessels. These findings suggest a tightly controlled regulation of FasL expression by P-EV, thereby indicating its potential to enhance effector T cell infiltration.

It is noteworthy that besides inactivating CD8^+^ tumor-infiltrating lymphocyte (TIL), vessel abnormalities in the TME also contribute to recruit immunosuppressive cells like Tregs [7]. Thus, we examined the intratumoral populations of CD8^+^ TILs and CD4^+^FoxP3^+^ Tregs (Fig. 6E). CD8^+^ TILs were rarely found in both DPBS and P-EV groups, whereas those in the P-EV+aPD-1 group were highly abundant, even compared to the aPD-1 group. Moreover, the intratumoral populations of CD4^+^FoxP3^+^ Tregs were significantly reduced in mice receiving P-EV+aPD-1, as compared to those treated with aPD-1 alone or DPBS.

## Discussion

Extensive research has been conducted on combination therapy strategies that integrate traditional cancer treatments, such as radiotherapy [28], photodynamic therapy [29], and chemotherapy [30], to enhance the efficacy of ICIs [31]. While these combinatorial approaches have demonstrated improved antitumor effects, concerns regarding toxicity and safety persist [32]. Consequently, there has been a paradigm shift toward strategies that modulate the TME to reinstate the cancer-immunity cycle [33]. This shift is crucial, as the immunosuppressive components within the TME significantly contribute to resistance against current ICIs, particularly in solid tumors [34].

Characterized by abnormal vasculature, hypoxia, and immunosuppressive stromal cells, the TME presents substantial obstacles to the success of cancer immunotherapy. As such, it has become a critical target for combination therapies aimed at overcoming these barriers. To address these challenges, various biomaterials have been developed to modulate immune responses and enhance therapeutic efficacy. Nanomaterials, in particular, have shown considerable promise as immunotherapeutic agents by inducing immunogenic cell death in cancer cells, thereby activating and proliferating immune cells [35–38]. Moreover, specific nanomaterials can be engineered to influence the behavior of immune cells, including dendritic cells and macrophages, thereby enhancing antitumor immunity [39–41]. Among the diverse array of nanomaterials, EVs offer distinct advantages due to their lower immunogenicity and reduced toxicity compared to synthetic materials [42].

In our previous studies, various strategies using EVs have been explored to augment the antitumor immunity. Representative approaches include increasing the immunogenicity of tumor exosomes through preconditioning [43] and reducing resistance to immune checkpoint therapy by inhibiting immunosuppressive tumor exosomes [44]. However, these methods primarily focus on modulating immune responses and may not fully address the physical and hypoxic barriers within the TME that hinder effective therapeutic outcomes. In this study, we present a novel strategy to improve therapeutic responsiveness of ICIs by targeting and modulating one of the primary physical barriers within the TME—the tumor vasculature, thereby improving the infiltration of effector T cells.

Owing to its feature as the physical and functional barrier to the infiltration and activity of effector T cells, the abnormal tumor vasculature has been considered one of therapeutic targets in cancer immunotherapy [45]. A growing body of evidence suggests that antitumor immunity can be revitalized by normalizing the tumor-associated vasculature via rebalancing proangiogenic factors and antiangiogenic factors. In this study, we explored the combined effect of an aPD-1 and an antiangiogenic P-EV to overcome the limitations of current immunotherapies by dysfunctional vasculature.

Despite their low PEDF content, P-EVs of this study have exhibited potent antiangiogenic effects, comparable to PEDF-H (Figs. 3 and 4). The observed reduction in migration and proliferation of endothelial cells, accompanied by disrupted network formation, suggests that P-EVs effectively suppress the angiogenic cascade essential for tumor growth and metastasis. Also, in vivo experiments further confirm that P-EVs harness the antiangiogenic properties of PEDF to inhibit tumor growth and ameliorate hypoxia within the TME. These results might be due to the transient normalization of tumor vasculature reported with traditional antiangiogenic therapies, which improve perfusion and reduce hypoxia [5].

The comparable therapeutic efficacy of P-EVs and PEDF-H highlights the potential of EVs as carriers for PEDF. It has been reported that several cytokines can be delivered bound to the EV surface [46]. This association of a secreted protein with the EV membrane has been shown to prolong and enhance efficacy, even when the proteins are exposed [47]. Similarly, the localization of PEDF on the EV surface may enhance interactions with endothelial receptors, potentially leading to superior biological responses compared to its soluble forms [24]. Moreover, the small EV formulation likely protects free soluble PEDF from proteolytic degradation and maintain protein conformation, thereby preserving its bioactivity under the challenging conditions of TME [24]. This implies that EV-mediated delivery systems not only improve in vivo stability and efficacy of therapeutic proteins such as PEDF but also reduce the required dosage. Given that PEDF functions as a negative regulator working through various mechanisms, its mode of action differs from simple antigen masking [22]. Thus, P-EVs can serve not only as a viable alternative strategy to overcome the limitations posed by reduced efficacy due to multiple resistance mechanisms in anti-VEGF/VEGF receptor antibody therapies [16,48], which are representative antiangiogenic treatments, but also potentially as a complement to enhance their therapeutic outcomes.

The combination of P-EV with aPD-1 therapy has demonstrated significant potential to enhance antitumor efficacy beyond that of checkpoint blockade alone. This synergistic effect stems from modulating capability of P-EV on the TME, particularly ascribed to its role in normalizing tumor vasculature and reducing hypoxia—a key factor in immunotherapeutic resistance. Furthermore, the observed decrease in FasL expression, mediated by P-EV treatment, could reduce the retention of immunosuppressive Tregs, while promoting infiltration of cytotoxic CD8^+^ T cells. Collectively, the results support that P-EV potently reshapes the TME and amplifies the antitumor response of the PD-1 blockade. In addition, the reduction in metastatic nodules signals that the antiangiogenic properties of P-EVs not only inhibit primary tumor growth but also prevent cancer cell dissemination.

Overall, given their unique features to modulate the TME, P-EVs might have the potential for applications in various combination treatment regimens such as cancer immunotherapy and conventional chemotherapy to counteract the multifaceted mechanisms of tumor progression and resistance. Future studies may focus on enhancing the tumor-targeting capabilities of P-EVs through advanced strategies such as the introduction of various ligands (i.e., antibodies, aptamers, peptides, etc.) [49,50]. These approaches could substantially improve their therapeutic outcomes and represent a promising direction for advancing the application of P-EVs in cancer treatment.

## Data Availability

The datasets used and/or analyzed during the current study are available from the corresponding author on reasonable request.
