# Supplementary figures and images for: PEDF-Enriched Extracellular Vesicle for Vessel Normalization to Potentiate Immune Checkpoint Blockade Therapy
Source: Biomater Res. 2024 Oct 1;28:0068. doi: 10.34133/bmr.0068 (PMC11443973; doi:10.34133/bmr.0068)

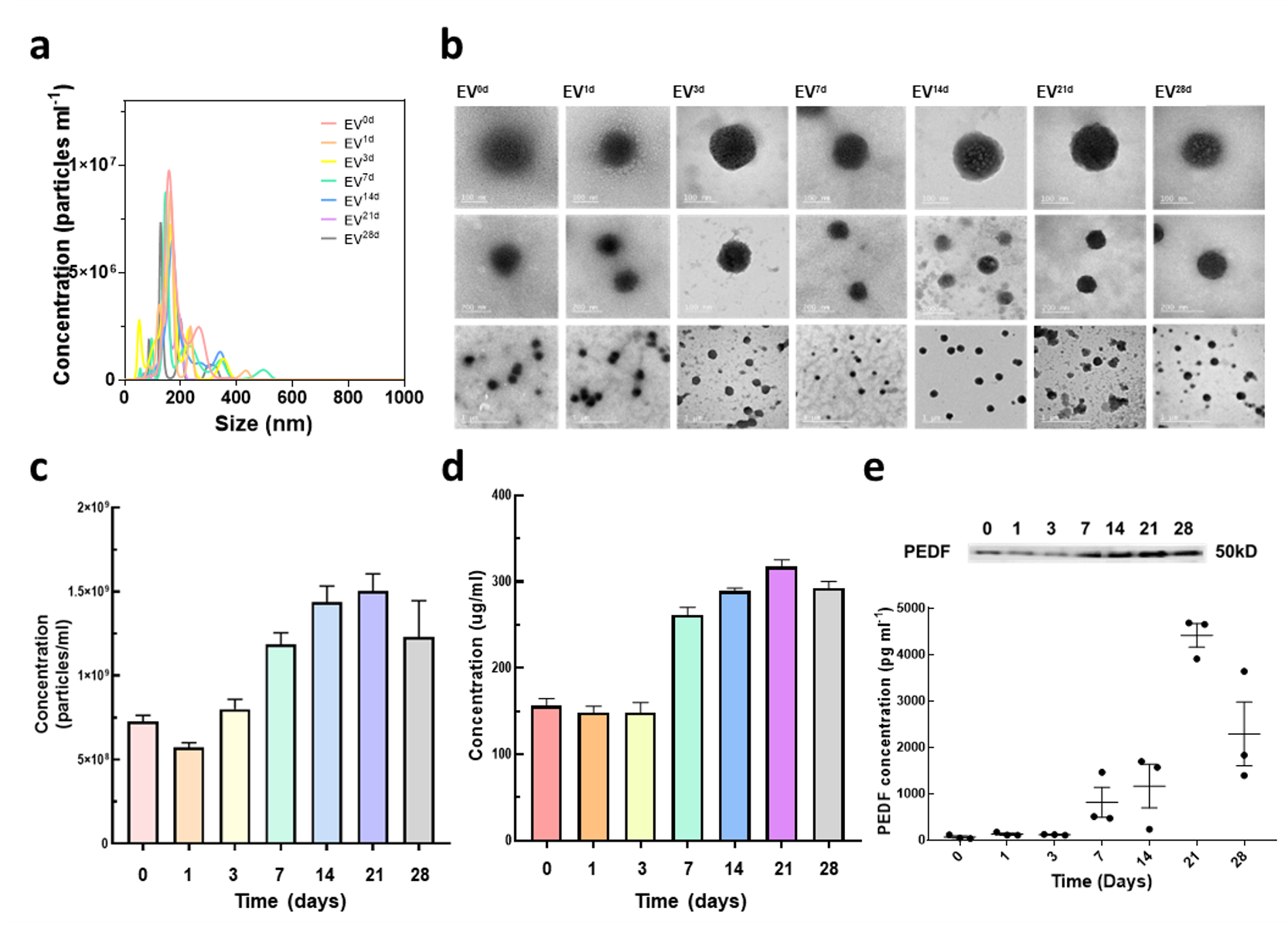

Supplement: Supplementary 1 — Figs. S1 to S5 [file bmr.0068.f1.zip › S1.png]

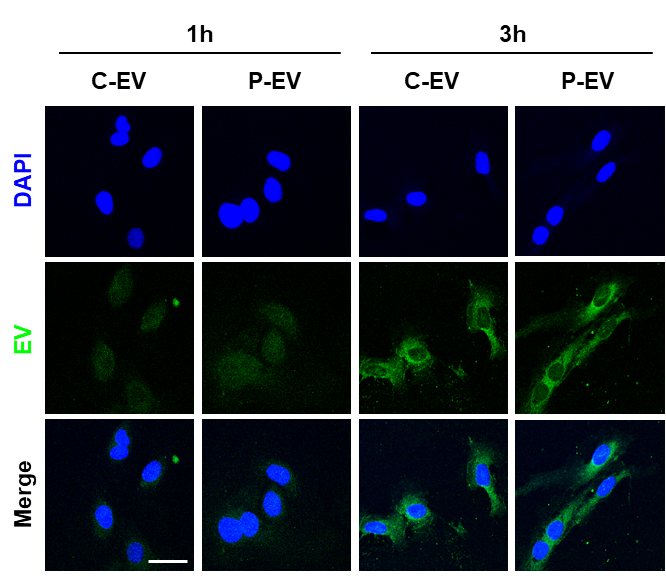

Supplement: Supplementary 1 — Figs. S1 to S5 [file bmr.0068.f1.zip › S2.png]

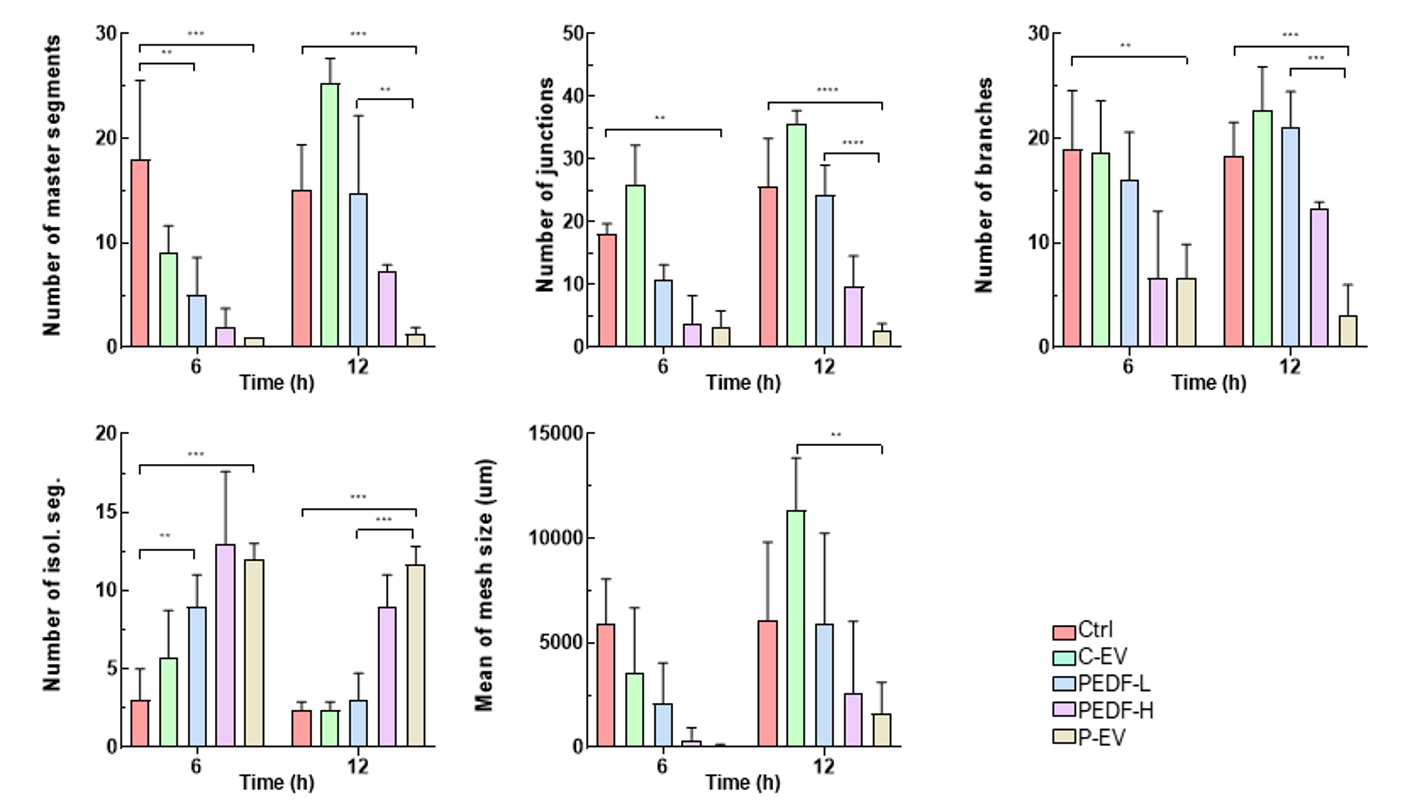

Supplement: Supplementary 1 — Figs. S1 to S5 [file bmr.0068.f1.zip › S3.png]

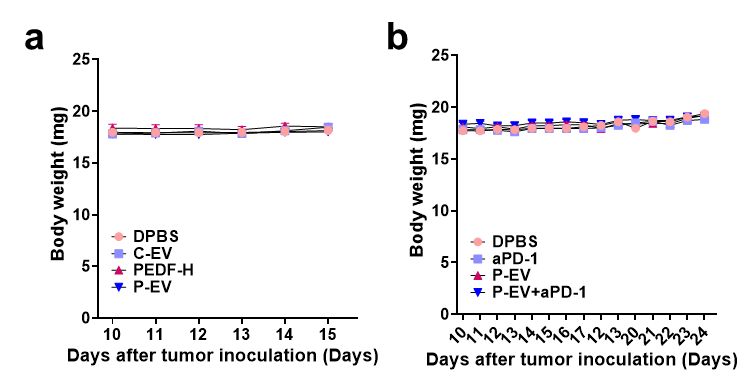

Supplement: Supplementary 1 — Figs. S1 to S5 [file bmr.0068.f1.zip › S4.png]

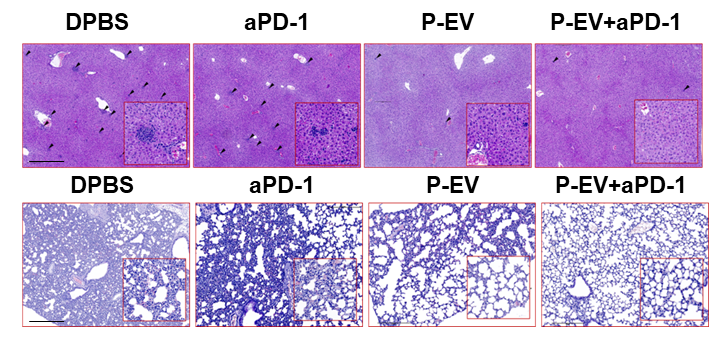

Supplement: Supplementary 1 — Figs. S1 to S5 [file bmr.0068.f1.zip › S5.png]
